# 

**Published:** 1834-01-01

**Authors:** 


### CONTENTS

OF THE

### MEDICAL QUARTERLY REVIEW,

### No. II. JANUARY 1, 1834.

---

### REVIEWS.

I.

Surgical Essays; the Result of Clinical Observations made at Guy's Hospital. By B. B. Cooper, F.R.S. . . . 229

II.

Précis Elémentaire de Physiologie. Par F. Magendie. . 245

III.

Alphabet of Scientific Chemistry, for the Use of Beginners. By James Rennie, M.A. . . . . 256

IV.

Surgical Observations on the Restoration of the Nose; and on the Removal of Polypi and other Tumours from the Nostrils. From the German of Dr. Dieffenbach, of Berlin. With the History and Physiology of Rhinoplastic Operations, Notes, and additional Cases. By John Stevenson Bushnan, F.L.S., M.R.C.S.E., &c. 264

V.

Observations on Obstetric Auscultation, with an Analysis of the Evidences of Pregnancy, and an Inquiry into the Proofs of the Life and Death of the Fœtus in Utero. By Evory Kennedy, M.D., Licentiate of the King and Queen's College of Physicians in Ireland, Lecturer on Midwifery, &c. With an Appendix, containing Legal Notes, by John Smith, Esq., Barrister at Law. . . 272

VI.

Medico-Chirurgical Transactions, published by the Medical and Chirurgical Society of London. Vol. XVIII. Part. I. . 278

- Dr. Bright on Disease of the Pancreas and Duodenum. 280
- Mr. Lloyd's Case of Jaundice. . . . 286
- Dr. Elliotson on the Discharge of Fatty Matters. . 288
- Mr. Arnott on Œsophagotomy. . . 290
- Mr. Langstaff on Medullary Sarcoma, &c. . . 291
- Mr. Stanley's Case of Bony Union, &c. . . 292

VII.

Clinical Observations on the Constitutional Origin of the various Forms of Porrigo. By George Macilwain. . . 293

VIII.

Lectures on the Diseases of the Urinary Organs. By B. C. Brodie, F.R.S. . . . . . . 297

*Reviews continued.*

IX.

Hunterian Reminiscences; being the Substance of a Course of Lectures on the Principles and Practice of Surgery, delivered by the late Mr. John Hunter, in the year 1785; taken in Shorthand, and afterwards fairly transcribed, by the late Mr. James Parkinson. Edited by his Son, J. W. K. Parkinson, m.r.c.s.l. . 307

X.

Transactions of the Medical and Physical Society of Calcutta. Vol. vi. 323
Mr. Raleigh on Couching. . . . 325
Mr. Bramley on Bronchocele, &c. . . . 327
Mr. Twining on some of the Effects of Iodine . . 328
Dr. Murray's Case of Traumatic Tetanus, &c. . . 329

XI.

The London Practice of Midwifery; including the most important Diseases of Women and Children. Chiefly designed for the Use of Students and Early Practitioners. The Sixth Edition, with Alterations, additional Practical Remarks, and new Sections. By Geo. Jewel, m.d. &c. . . . . . 331

XII.

The Edinburgh Medical and Surgical Journal, No. cxvii. . 335
Dr. Ogston on Intoxication, &c. . . . 336
Dr. Coldstream on the Climate of Torquay, &c. . 337
Mr. Keith and Mr. Liston on Lithotrity . . 338

XIII.

The Origin and Progress of the Malignant Cholera at Manchester. By Henry Gaulter, m.d. . . . . 341

XIV.

Introduction to the Study and Practice of Midwifery, and the Diseases of Women and Children. By W. Campbell, m.d. . . 348

XV.

A new Method of making Anatomical Preparations. By Joseph Swan 355

XVI.

The Hand, its Mechanism, and Vital Endowments, as evincing Design. By Sir Charles Bell, k.g.h., &c. . . . 358

XVII.

The History of a Case in which Animals were found in Blood drawn from the Veins of a Boy; with Remarks. By J. S. Bushnan, f.l.s. . 364

XVIII.

The Cyclopædia of Practical Medicine.
Dr. Montgomery on the Signs of Pregnancy and Delivery 366
Dr. Beatty on Rape, &c. . . . . 367

XIX.

Chemical Diagrams, &c. By Alexander Lee, a.m. . . 369

XX.

Syllabus of a Course of Lectures on Surgery. By F. Tyrrell, Esq. . 370

XXI.

Facts establishing the Deleterious Properties of Rice, &c. By Dr. Tytler 371

*Reviews continued.*

XXII.
Illustrations of the Effects of Poisons. By G. LEITH ROUPEL, M.D. . 371

XXIII.
A Dictionary of Practical Medicine. By JAMES COPLAND, M.D. 372

XXIV.
Principles and Illustrations of Morbid Anatomy. By J. HOPE, M.D. 372

XXV.
A Rational Exposition of the Physical Signs of the Diseases of the Lungs and Pleura, &c. By CHARLES J. B. WILLIAMS, M.D. . . ib.

XXVI.
On the Study of Medicine. By ROBERT E. GRANT, M.D. . . 373

XXVII.
Thoughts on Medical Reform. By A RETIRED PRACTITIONER . ib.

XXVIII.
A Series of Anatomical Plates, in Lithography. Edited by Dr. QUAIN 374

XXIX.
The Gardener's Dictionary. By PHILIP MILLER, F.R.S. . . 375

### Original Communications.

I.
Cases extracted, by permission, from the Note-books of HENRY DAVIES, M.D. . . . . . 376

II.
Heads of Clinical Observations on Mistakes and Oversights in the Treatment of Strangulated Hernia. By HERBERT MAYO, F.R.S. 379

III.
A Case of Peritonitis, with Fœcal Abscess, in which the Patient recovered; with Remarks. By JOHN BURNE, M.D. . . 385

IV.
A Case of Lithotomy by the High Operation; with Remarks. By C. A. KEY, Esq. . . . . . 388

V.
On the Removal of Morbid Enlargements of the Integuments of the Nose. By JOHN DALRYMPLE, Esq. . . . 395

VI.
Account of Two Cases of Phthisis, attended with peculiar Circumstances. Communicated to the Harveian Society, by W. STROUD, M.D. 401

VII.
Cases of Ligature of the Common Carotid Artery. By H. MAYO, F.R.S. 410

VIII.
On Inflammatory and Spasmodic Strictures of the Urethra. By FRED. TYRRELL, Esq. . . . . . 414

IX.
Case of Empyema. Communicated by JOHN BULLOCK, Esq. . 423

X.
Case of Recovery from an Abdominal Abscess. By J. VALENTINE, Esq. 425

### COLLECTANEA.

### PATHOLOGY AND PRACTICE.

Remarkable Case of Ascites . . . . 427

On the Use of the Sulphate of Cadmium in Diseases of the Eye . 428

Sudden Complete Amaurosis, from Violent Inflammatory Attack. 430

Complete Amaurosis, with Congestion in the Head. . 431

Post-mortem Convulsions in Cholera Patients. . . 432

Treatment of Hydrophobia. . . . . 433

Case of Erysipelas of the Face . . . 435

Effects of an Obturator made of different Metals . . 436

Bad Rice the supposed Cause of Asiatic Cholera . . ib.

A. L. Wigan and the Ringworm . . . 437

Binelli's Solution and Kreosot . . . 438

Treatment of Cholera by Calomel alone. Cases of Climacteric Decay 440

Post-mortem Examination of a Painter. The Cretins of Salzburg 441

Steel Busks. Case of Apoplexy . . . 442

Medicated Baths . . . . . 443

Treatment of Ascites. Transference of Vital Power . . 444

Convulsions accompanying the Cutting of Second Teeth . . 445

A Case of Anastomosing Aneurism of the External Maxillary Artery . 447

### MISCELLANEOUS.

Statistics of European Mortality . . . . 448

St. Vitus's Dance. Prometheus a Distiller . . . 449

On the Operation of the Anatomy Act, by Dr. Somerville . 450

The Date Palm . . . . . 452

Order of the Eruption of the First Teeth . . 453

English Eye-Surgery, a Sketch; by Professor Walther, of Munich 454

Anecdote of the Domestic Cat. Difficulties overcome by Science 458

Cost of becoming a Man of Letters. Dr. Thomas Young . 459

Hottentot's Bread. Arboreous Ferns . . . 460

Easy Parturition among Savages . . . . 461

Rapid Parturition, as connected with Forensic Medicine . 462

Annual Bills of Mortality . . . . 463

### Medical Politics and Intelligence.

1. The Proposed Scotch Ascendancy in the College of Physicians . 464

2. The Examination at Apothecaries' Hall . . . 465

3. Dispensaries . . . . . . 467

4. Medical Society of London . . . . 468

### OBITUARY.

Dr. Milligan . . . . . . 471

Meteorological Register. Notices . . . 472

---

**Advertising Sheet—(see End of the Number.)**

